# Transforming into a patient-centered medical home: Understanding facilitators, barriers and context from a synthesis of implementation studies

**DOI:** 10.1186/1748-5908-10-S1-A68

**Published:** 2015-08-20

**Authors:** Sarah J Shoemaker

**Affiliations:** 1 Health Policy, Abt Associates Inc., Cambridge, MA 02139, USA

## Objective

To understand the facilitators, barriers and context that were present in transformations to patient-centered medical homes (PCMH).

## Methods

A meta-synthesis of qualitative findings from implementation evaluations and experiences of adult PCMH transformation in pilots/demonstrations, including national, multi-state and single state demonstrations. The majority of practices represented family or internal medicine with an average of 14.1 practices per demonstration (range: 1-36). Most were mixed-methods evaluations and included: site visits, observations, interviews, and focus groups. The mean number of interview respondents was 64.5.

## Findings

Qualitative findings from 12 implementation studies of PCMH transformations were synthesized. The barriers and challenges encountered in PCMH transformation included: staffing challenges related to limited time and role restructuring like team-based care; financial imitations and/or limited support; hierarchical leadership or not believing in transformation; electronic health records are not a 'plug and play'; change fatigue is a serious concern, even for highly-motivated practices; and in safety net systems patient demand for services and deficient linkages to hospitals/health-systems. It was clear from the studies that becoming a PCMH requires true transformation and is more than just a series of changes, it requires substantial shift in roles and mental models and can benefit from change management, thus an incremental approach can be effective. Motivation and commitment were important facilitators. Additional lessons includes: the requirement of a team-based approach; practice adaptive reserve is critical to manage change; leadership believing in transformation, engaging staff and being adaptive; and practice facilitation and learning appear to be effective strategies to support PCMH transformation.

## How research advances the field of D&I

Understanding the implementation strategies that were effective in the transformation of PCMHs from across demonstrations and pilots can advance the understanding of how to support uptake of other PCMHs and future health care redesign efforts.

